# LEA29Y expression in transgenic neonatal porcine islet-like cluster promotes long-lasting xenograft survival in humanized mice without immunosuppressive therapy

**DOI:** 10.1038/s41598-017-03913-4

**Published:** 2017-06-15

**Authors:** L. Wolf-van Buerck, M. Schuster, F. S. Oduncu, A. Baehr, T. Mayr, S. Guethoff, J. Abicht, B. Reichart, N. Klymiuk, E. Wolf, J. Seissler

**Affiliations:** 1 Medizinische Klinik und Poliklinik IV, Diabetes Zentrum - Campus Innenstadt, Klinikum der Ludwig-Maximilians-Universität, München, Germany; 2 Medizinische Klinik und Poliklinik IV, Division Hematology and Oncology, Klinikum der Ludwig-Maximilians-Universität, München, Germany; 3 Chair for Molecular Animal Breeding and Biotechnology, and Laboratory for Functional Genome Analysis (LAFUGA), Gene Center, Ludwig-Maximilians-Universität, München, Germany; 4 Department of Anesthesiology, Walter-Brendel-Zentrum, Ludwig-Maximilians-Universität, München, Germany; 5 Department of Cardiac Surgery, Ludwig-Maximilians-Universität, München, Germany; 6 German Center for Diabetes Research (DZD), München-Neuherberg, Germany

## Abstract

Genetically engineered pigs are a promising source for islet cell transplantation in type 1 diabetes, but the strong human anti-pig immune response prevents its successful clinical application. Here we studied the efficacy of neonatal porcine islet-like cell clusters (NPICCs) overexpressing LEA29Y, a high-affinity variant of the T cell co-stimulation inhibitor CTLA-4Ig, to engraft and restore normoglycemia after transplantation into streptozotocin-diabetic NOD-SCID IL2rγ^−/−^ (NSG) mice stably reconstituted with a human immune system. Transplantation of *INS*LEA29Y expressing NPICCs resulted in development of normal glucose tolerance (70.4%) and long-term maintenance of normoglycemia without administration of immunosuppressive drugs. All animals transplanted with wild-type NPICCs remained diabetic. Immunohistological examinations revealed a strong peri- and intragraft infiltration of wild-type NPICCs with human CD45^+^ immune cells consisting of predominantly CD4^+^ and CD8^+^ lymphocytes and some CD68^+^ macrophages and FoxP3^+^ regulatory T cells. Significantly less infiltrating lymphocytes and only few macrophages were observed in animals transplanted with *INS*LEA29Y transgenic NPICCs. This is the first study providing evidence that beta cell-specific LEA29Y expression is effective for NPICC engraftment in the presence of a humanized immune system and it has a long-lasting protective effect on inhibition of human anti-pig xenoimmunity. Our findings may have important implications for the development of a low-toxic protocol for porcine islet transplantation in patients with type 1 diabetes.

## Introduction

Transplantation of isolated islets or whole pancreas are currently the only treatment options to restore normal glucose homeostasis in patients with type 1 diabetes. The success of islet cell transplantation is limited by the shortage of organ donors, the need for transplantation of a large number of islets, and by side effects of immunosuppressive drugs^1^. Transplantation studies in non-human primates have shown that porcine islets are a promising alternative, almost unlimited cell source but face the problem of a strong xenoreactive rejection that requires an intensive immunosuppressive regimen with severe side effects, which impedes clinical application^2–6^. Systemic blockade of costimulatory pathways CD80/86-CTLA-4 and CD40-CD154 has emerged as a promising strategy to achieve control of allogeneic and xenogeneic graft rejection but is so far not applicable in clinical practice^7, 8^. We aimed to develop a method for local immunomodulation after islet xenotransplantation by transgenic (tg) overexpression of immunomodulatory molecules in porcine beta cells. Recently, our group generated a novel transgenic (tg) pig line expressing the CTLA-4Ig analogue LEA29Y under the control of the porcine insulin (*INS*) gene promoter. We demonstrated that transplanted *INS*LEA29Y-tg porcine neonatal porcine islet-like clusters (NPICCs) display normal beta cell function and are protected from rapid T lymphocyte-mediated rejection in NOD-SCID IL2rγ^−/−^ (NSG) mice after the transfer of human PBMCs^9^. In this model, however, the observation period was restricted to 30 days due to the development of graft-versus-host disease and, therefore, the long-term effect of LEA29Y expression on xenograft rejection as well as the potential of NPICCs to engraft in the presence of an active human immune system remained unknown.

In the present study, we evaluated grafting and survival of *INS*LEA29Y-tg NPICCs in a humanized mouse model that allows both investigation of *INS*LEA29Y-tg NPICC engraftment and survival in the presence of a functional human immune system and long-term analysis of human anti-pig immune rejection.

## Results

### Reconstitution of human immune cells in NSG mice and immunological monitoring

**Figure 1:**
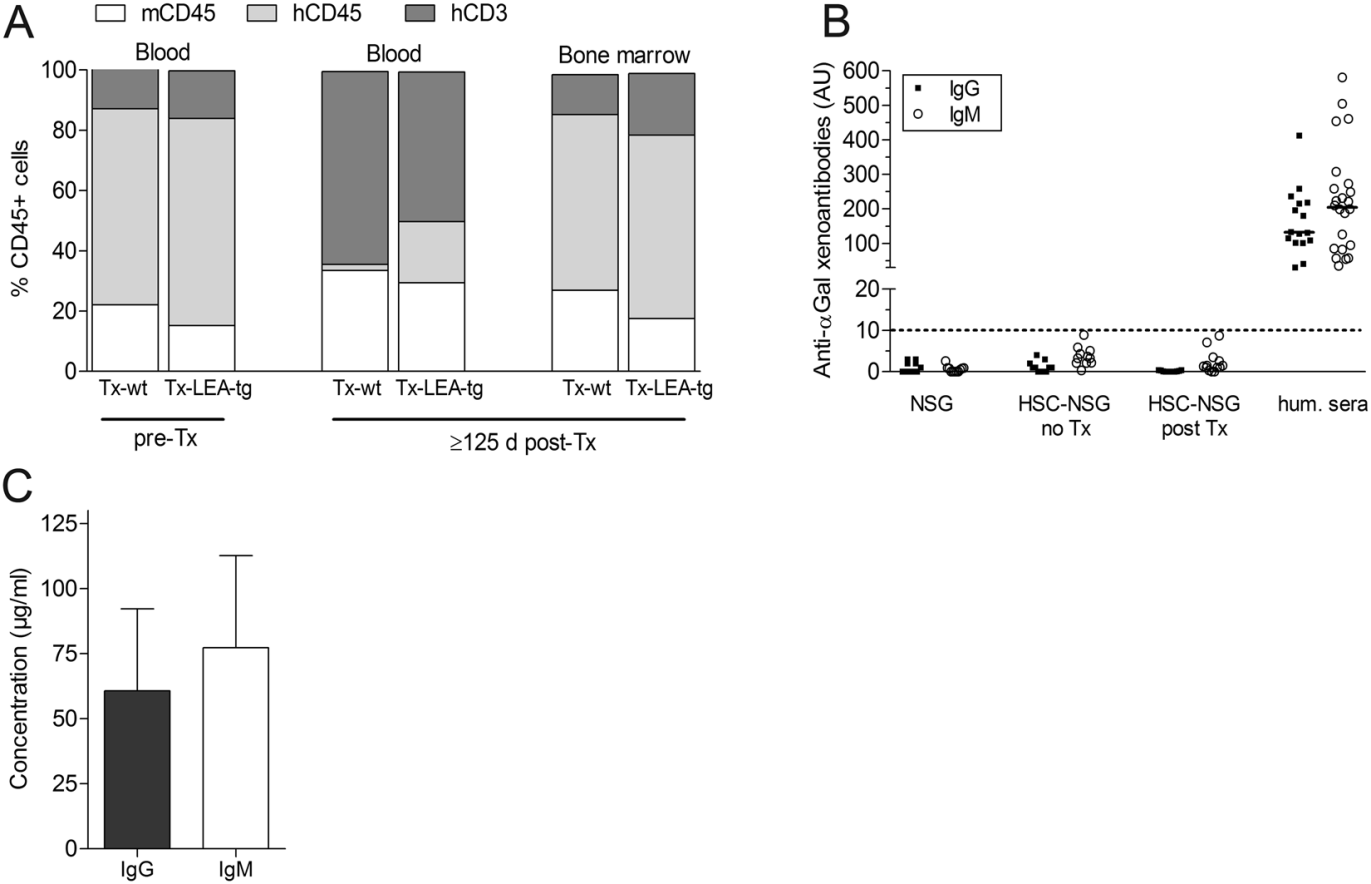
Reconstitution of human immune system and production of antibodies in NSG mice (HU-SRC-SCID) transplanted with wild-type (Tx-wt) or *INS*LEA29Y transgenic (Tx-LEA-tg) NPICCs. Isolated human CD34^+^ hematopoietic stem cells were transferred to NSG mice. After 16–20 weeks and at the end of the observation period the percentage of human immune cells (hCD45^+^) and immune cell subsets (gated on human CD45) were analyzed by FACS (**A**). Xenoantibodies (**B**) and plasma levels of human IgG and human IgM (**C**) were measured by ELISA.

Percentage of human immune cells during follow-up was determined by FACS analysis at weeks 16–20 and at the end of the study to confirm stable reconstitution with a human immune system throughout the observation period. As illustrated in Fig. 1A, there was a high percentage of human immune cells at both time-points (82 ± 12.3 before and 68 ± 24% hCD45^+^ cells after NPICC transplantation (peripheral blood) and 78 ± 18% hCD45^+^ cells in bone marrow). Among CD3^+^ T lymphocytes the majority belong to the CD4^+^ population with a CD4/CD8 ratio of about 2.6 (before transplantation). There were no differences in the proportion of human CD45^+^, CD3^+^, CD4^+^, and CD8^+^ cells between different transplantation groups.

In addition, total plasma human immunoglobulin concentrations and the presence of xenoantibodies directed to the αGal epitope were analyzed at the end of the observation period. Significant plasma levels of human IgG (61 ± 31 µg/ml) and IgM (77 ± 35 µg/ml) were detectable. Concentrations of hIgM and hIgG were not different in animals transplanted with wild-type (wt) NPICCs and LEA29Y-tg NPICCs. Xenoantibodies directed to the αGal epitope were not detectable (Fig. 1B).

### Development of normoglycemia in transplanted mice

**Figure 2:**
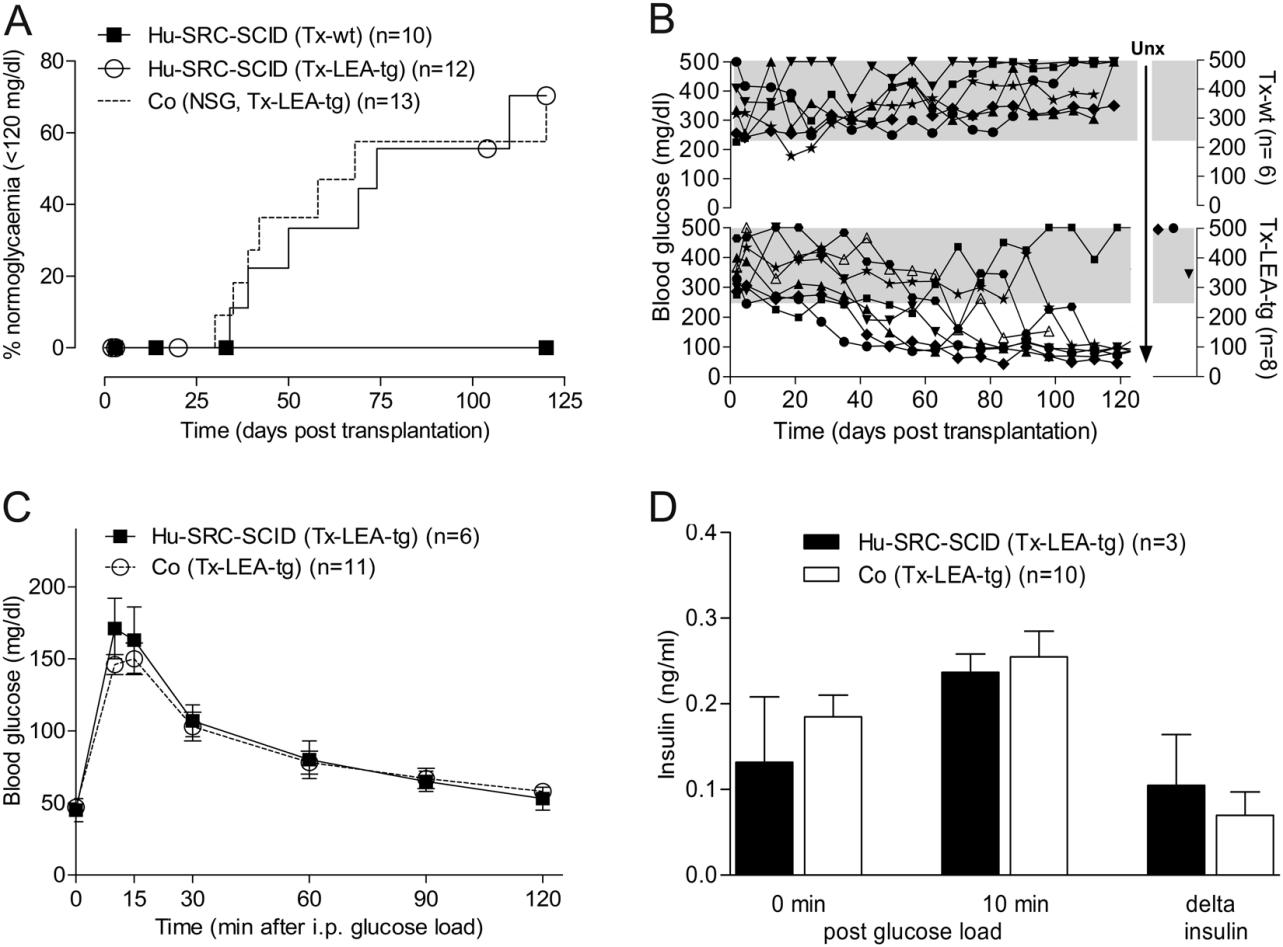
Transplantation of LEA29Y-tg neonatal porcine islet-like clusters (NPICCs) into diabetic NSG mice carrying a human immune system (HU-SRC-SCID). (**A**) Development of normoglycemia (random blood glucose levels consistently <120 mg/dl), blood glucose (**C**) response and insulin secretion (**D**) during intraperitoneal glucose tolerance testing was comparable in humanized Tx-LEA-tg and immunodeficient NSG mice transplanted with equivalent numbers of NPICCs. The 125-day time course of mice with LEA-tg NPICCs shows normalization of blood glucose levels in n = 5 mice, near normal glucose levels in n = 2 animals, and persistent hyperglycemia in n = 1 mouse (**B**). In contrast, all Tx-wt remained hyperglycemic (Log-rank test p = 0.039) (**A**,**B**). N = number of animals examined per group.

NPICCs require an *in vivo* maturation period of 2–4 months until normal beta cell function is achieved^9–11^. To determine the capacity of LEA29Y expression to inhibit human immune cell-mediated beta cell rejection NOD-SCID IL2rγ^−/−^ mice with >50% human CD45^+^ cells in peripheral blood (HU-SRC-SCID mice) were rendered diabetic by STZ treatment followed by transplantation with wt or LEA29Y-tg NPICCs. During follow-up of 4 months development of normoglycemia was observed in 70.4% HU-SRC-SCID mice transplanted with LEA29Y-tg NPICCs but in none of the animals transplanted with wt NPICCs (Fig. 2A,B) (p < 0.05). In the group transplanted with LEA29Y-tg NPICCs two additional mice developed near normal blood glucose levels (138–155 mg/dl) and only one mouse failed to improve hyperglycemia (Fig. 2B). As illustrated in Fig. 2B all mice transplanted with wt NPICCs showed persistent hyperglycemia requiring insulin treatment throughout the post-transplant period. The percentage of mice developing normal glucose homeostasis as well as time to reach normoglycemia after transplantation of LEA29Y-tg NPICCs was similar in HU-SRC-SCID mice (median 59.5 days; mean 62.7 ± 11.5 days) and in NSG mice which were not reconstituted with an immune system (grafting control NSG mice; median 42.0 days; mean 66.3 ± 13.5 days) (p = 0.86). Measurement of beta cell function in mice which achieved normoglycemia revealed normal glucose tolerance, similar blood glucose levels (area under the curve [AUC_glucose_] 10245 ± 1268 vs 9959 ± 583), and similar first phase insulin secretion (delta porcine insulin_0–10__min_, 105 ± 59 pg/ml vs 74 ± 32 pg/ml) in both transplantation groups (Fig. 2C). Removal of graft-bearing kidneys (n = 3 mice) resulted in rapid reoccurrence of diabetes (BG >350 mg/dl) indicating that the graft was responsible for normal glucose homeostasis (Fig. 2B). In the other mice mouse C-peptide levels were below the detection limit at the end of the observation period. In addition, only few if any residual beta cells were detected in immunohistochemical stainings of recipient pancreata in both transplantation groups excluding endogenous beta cell regeneration (Supplemental Figure 1). Mean plasma concentration of LEA29Y measured at the end of the study was 0.344 ± 0.039 µg/ml.

### Histological analysis of graft infiltrating cells

**Figure 3:**
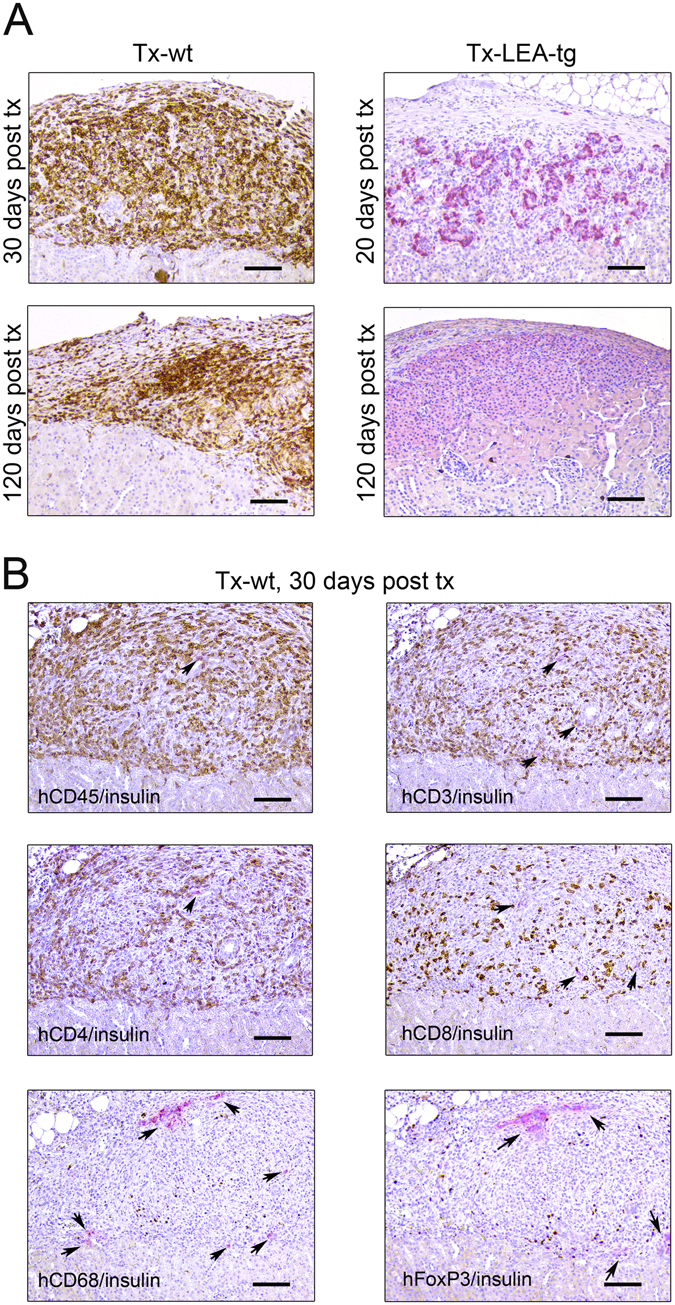
Histological analysis of transplanted NPICCs. Islet xenografts were investigated at day 30 and day 120 post Tx. (**A**) Staining for insulin (red) revealed that wild-type NPICCs were rejected and displayed a strong infiltration with human CD45^+^ (brown) immune cells. LEA29Y expressing islets were protected from rejection and exhibited a preserved islet xenograft with few infiltrating CD45^+^ cells. (**B**) Transplanted wild-type NPICCs at day 20–30 post Tx stained against insulin (red) and human CD45, CD3, CD4, CD8, CD68, and FoxP3 (brown): only few insulin^+^ cells (arrows) are detectable but massive immune cell accumulation whereby infiltrating cells mostly consist of hCD4^+^ and hCD8^+^ lymphocytes, some macrophages, and few FoxP3^+^ T cells. Scale bar: 100 μm.

**Figure 4:**
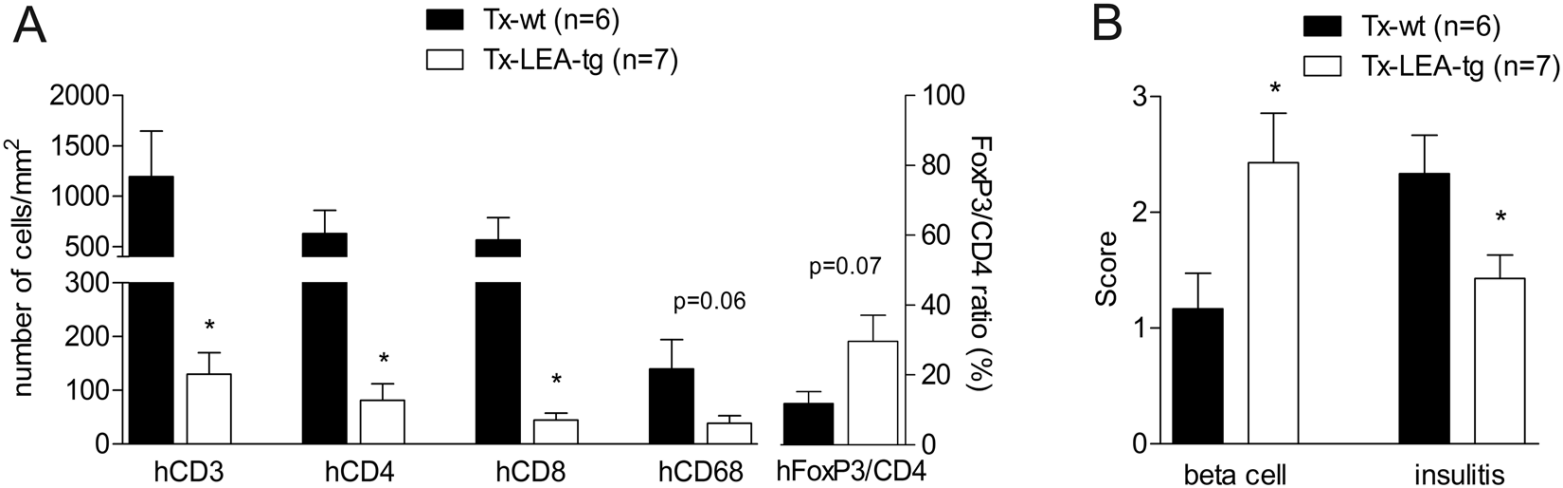
Histological scoring of NPICC grafts and quantification of immune cell infiltration. (**A**) The beta cell insulin score was significantly higher in Tx-LEA-tg as compared to Tx-wt, whereas the insulitis score and the number of infiltrating hCD3^+^, hCD4^+^, and hCD8^+^ T cells was significantly lower in these animals (p < 0.05) (**A**). There was a non-significant trend towards a higher frequency of hCD68^+^ macrophages and a lower FoxP3^+^/CD4^+^ ratio in animals transplanted with wt NPICCs. N = number of animals examined per group. ^*^p < 0.05 Tx-LEA-tg vs. Tx-wt.

Rejection of grafted porcine islets and NPICCs is associated with infiltration of innate and adaptive immune cells^2, 3, 5, 6, 9^. There was a massive peri- and intragraft infiltration with human CD45^+^ immune cells into wt NPICC grafts 3–4 weeks after transplantation (Fig. 3). Most of the infiltrating cells were T lymphocytes (hCD3^+^) consisting of CD4^+^ and CD8^+^ subpopulations. Additionally, some cells stained positive for hCD68 (macrophages) or FoxP3 (regulatory T cells) (Fig. 3B) were observable. As illustrated in Fig. 3A, well preserved, strongly insulin positive endocrine tissue with only few infiltrating hCD3^+^, hCD4^+^, hCD8^+^ T cells and hCD68^+^ macrophages was detected in the group transplanted with LEA29Y-tg NPICCs. NK cells (h) were not detected in the subcapsular grafts in both transplantation groups (Supplemental Figure 2). The grafts of the 2 mice which developed near normoglycemia did not differ histologically from the grafts of normoglycemic mice (Supplemental Figure 3A,B). The graft of the mouse that failed to develop normoglycemia after transplantation of LEA29Y-tg NPICCs showed no insulin staining and only few immune cells (beta cell insulin score: 0; insulitis score: 1) suggesting either failure of primary grafting or complete rejection (Supplemental Figure 3C). Quantification of immune cell infiltrates in both transplantation groups revealed a significant lower insulitis score in mice transplanted with LEA29Y-tg NPICCs (p < 0.05) (Fig. 4B). Figure 4A summarizes quantification of graft infiltrating immune cells. There were significantly higher numbers of T cells (hCD3^+^, hCD4^+^, hCD8^+^) per mm^2^ graft area and a trend towards an increased macrophage density in mice transplanted with wt NPICCs as compared to animals transplanted with LEA29Y-tg NPICCs (p < 0.05). To analyze whether FoxP3^+^ regulatory T cells are localized within the graft, the number of FoxP3^+^ cells was quantified and expressed in percent of the number of infiltrating CD4^+^ cells. The FoxP3^+^/CD4^+^ ratio was considerably higher in mice with LEA29Y-tg NPICCs but did not reach statistical significance (Fig. 4A). These data indicate that expression of LEA29Y in beta cells strongly modulates und inhibits cellular human anti-porcine xenorejection.

### Long-lasting islet xenograft survival

**Figure 5:**
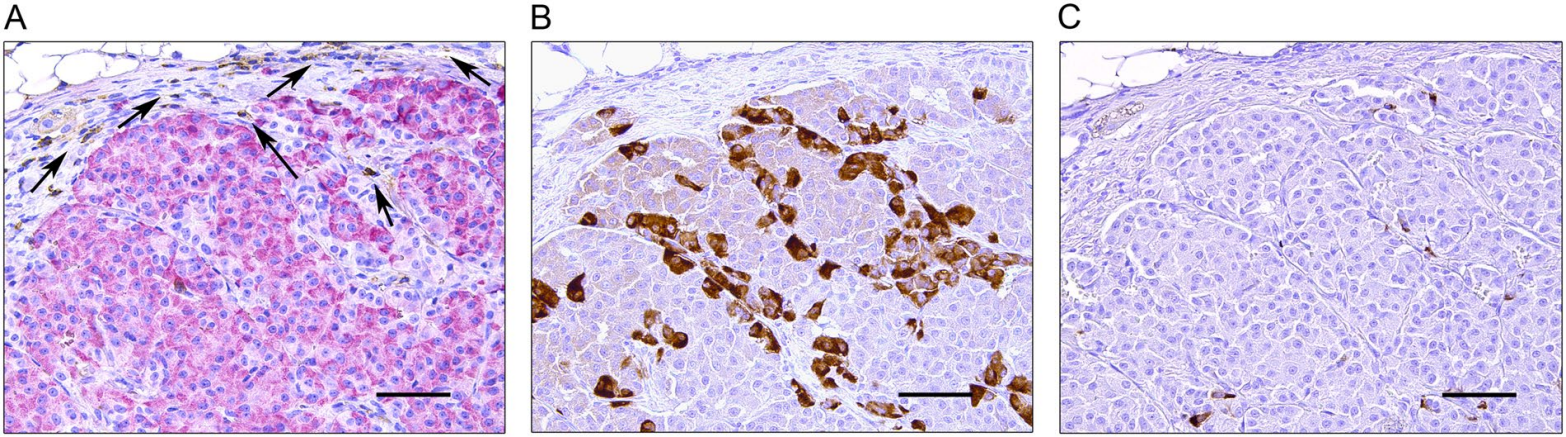
Long-term preservation of beta cell function in HU-SRC-SCID mice transplanted with LEA29Y expressing NPICCs. Xenograft was harvested 29 weeks post transplantation and stained for (**A**) human CD45 (brown) and insulin (red), (**B**) glucagon (brown) and (**C**) somatostatin (brown). Local LEA29Y expression preserved ICC structure and restricted human immune infiltration to the periphery of the transplantation site. Scale bar: 100 μm.

In two normoglycemic mice transplanted with LEA29Y-tg NPICCs the follow-up period was extended to >6 months (29 and 34 weeks). Both mice exhibited persistent normoglycemia until the end of the study and a normal glucose tolerance. As illustrated in Fig. 5 the architecture of transplanted islets was well maintained and there were only single hCD45^+^ immune cells in the periphery of the grafted tissue. Thus, local LEA29Y expression has a long-lasting protective effect without the need of systemic immunosuppression.

## Discussion

The strong xenogeneic immune rejection is the major obstacle in pig-to-human islet transplantation. The striking advances in the generation of knock-out and transgenic pigs suggest that it may be possible to overcome the major immunological barriers within the next years^12^. Our approach is to overexpress immunomodulatory molecules in beta cells to provide a local immunosuppressive environment at the transplantation site. The present study demonstrated for the first time long-lasting survival of LEA29Y-tg porcine beta cells for more than 6 months in NSG mice carrying a stable human immune system without administration of additional immunosuppressive therapy.

Preclinical studies in non-human primates (NHP) reported on insulin independence and survival of transplanted wt porcine islets or islets expressing hCD46 using induction therapy with T cell depleting antibodies and high dose maintenance treatment with several combinations of immunosuppressive drugs^2, 3, 5, 6, 13, 14^. All successful protocols included drugs blocking the second costimulatory signal in the interaction between antigen presenting cells (APS) and T cells. It is known that xenoimmunity is particularly dependent on CD80/86-CTLA-4 and CD40-CD154 signalling^15, 16^. Blocking the CD80/CD86 pathway by systemic administration of CTLA-4Ig or LEA29Y has been shown to efficiently inhibit effector T cell activation and proliferation, reduce porcine islet rejection, and does not adversely affect beta cell function^2, 5, 6, 13, 14^. In addition, prolonged survival of adenoviral vector transduced pig islets expressing porcine CTLA-4Ig has been reported^17^.

In the present study we used neonatal porcine islet-like cluster because NPICCs are thought to have some advantages over adult islets: 1) the isolation process of NPICCs is much easier and results in isolates of reproducible quality, 2) NPICCs are more robust against hypoxia and inflammation and breeding of transgenic piglets is much less expensive^10, 18^. This latter point may be of particular importance with regard to the translation of this cell therapy into the clinic. However, NPICCs are immature and require several weeks until insulin response to glucose is fully established^10, 19^. This limitation will be no major hurdle for future clinical application since T1D patients are on insulin treatment and can adapt insulin dose during the maturation period.

In a previous study we showed that LEA29Y-tg NPICCs were protected from rejection after transfer of human PBMCs indicating inhibition of T cell-mediated rejection^9^. In this model the follow-up period is restricted to about 30 days due to the development of a severe graft-versus host disease (GvHD). To overcome these limitations we here used an improved mouse model lacking GvHD, where the immune system is established by CD34^+^ hematopoietic stem cell transfer several months before islet cell transplantation. The HU-SRC-SCID model has been successfully used to establish mice with human T cells, B cells, macrophages, and dendritic cells^20–23^. It has the advantage that it more closely resembles the clinical situation and allowed us to perform long-term monitoring of NPICC engraftment and rejection. We here demonstrate that HU-SRC-SCID mice are able to recognize transplanted wt NPICCs, mediate a significant anti-porcine immune response with human CD45^+^ leukocyte infiltrates, and are capable to infiltrate and destruct the wt xenograft. This provides evidence that HU-SRC-SCID mice are able to develop a functional active adaptive immune response.

Overexpression of LEA29Y in porcine beta cells had a profound inhibitory effect on cellular rejection over several months. Consistent with the metabolic data we observed suppression of peri- and intragraft infiltrations with human T cells and macrophages at early and late post-transplant stages. The reduced number of CD4^+^ and CD8^+^ T cell infiltrates in LEA29Y-tg grafts indicates that transplanted LEA29Y-tg NPICCs can prevent the activation and recruitment of effector T cells to the transplantation site leading to long-term protection from rejection in the presence of a human immune system. The identification of an increased ratio of FoxP3^+^/CD4^+^ cells in LEA29Y-tg grafts suggests that regulatory T cells may be involved in the prevention of xenograft rejection. This interpretation is consistent with a recent publication reporting on FoxP3^+^ Treg cell-mediated tolerance induction against transplanted wild-type NPICCs by short-term treatment of BALB/c mice with CTLA-4 Fc and anti-CD154 antibodies^24^. Since transgenic grafts showed a tendency towards an increased proportion of intragraft FoxP3^+^ cells in the present study, the elucidation of the exact mechanisms involved in the protection of LEA29Y expressing xenografts requires further investigation.

FACS analysis carried out at the end of the study confirmed stable maintenance of human immune cells in all mice. Importantly, we observed neither increased susceptibility to infections in mice with LEA29Y-tg NPICCs nor significant differences in the proportion of human leukocytes and CD4^+^/CD8^+^ T cell populations between the transplantation groups. Additionally, plasma concentrations of LEA29Y were 30–100-fold lower as described in studies using belatacept for systemic immunosuppression after organ transplantation^25^. These findings suggest that beta cell-specific LEA29Y expression leads to high local but low systemic LEA29Y levels that may not have a strong systemic immunosuppressive effect^9^.

Naturally occurring antibodies directed to the αGal epitope are the major xenoantibodies in human sera and are induced after transplantation of porcine tissue in human beings and non-human primates^14, 26^. Because αGal Abs may be involved in xenorejection of NPICCs^13^, we measured total human IgG/IgM plasma concentrations and αGal Abs. In agreement with previous studies, HU-SRC-SCID mice produced low, but clearly detectable levels of human IgM and IgG antibodies, which correspond to about 5% (IgM) and 0.5% (IgG) of levels present in normal human plasma, respectively^27, 28^. IgG and IgM αGal Abs were not detected suggesting that αGal xenoantibodies are not involved in the rejection of wt NPICCs in the present study, but their relevance in a clinical setting needs to be addressed by using available knockout pigs (GTKO)^29^.

Although NSG mice allow high-level engraftment of human hematopoietic stem cells, the limitation of the present study is that these mice do not achieve a completely normal human immune system. The HU-SRC-SCID model shows decreased maturation of B cells and impaired innate immune cell development, e.g. reduced monocyte and NK cell activation^23, 27, 30^. This is explained by the lack of reactivity between murine cytokines and human immune cells. Consistently, we did not identify CD56^+^ NK cells infiltrating the grafts of transplanted mice. Therefore, it may be difficult to extrapolate our findings directly to the clinical situation and further preclinical studies in diabetic non-human primates are required.

In conclusion, we here demonstrated that beta cell-specific expression of LEA29Y is effective in long-lasting inhibition of xenoreactive, human immune cell-mediated rejection. Thus, overexpression of LEA29Y in porcine islets is very promising to develop a low-toxic immunosuppressive protocol for xenotransplantation.

## Methods

### Ethical statement

The study was approved by the university ethics committee and was performed according to the principles of the Declaration of Helsinki. All animal experiments were conducted in accordance and with the approval of the local animal welfare committee (district government of Upper Bavaria).

### Animals

NOD-SCID IL2rγ^−/−^ (NSG) mice, which lack mature T-, B-, and NK cells, were obtained from The Jackson Laboratory (USA) and housed under standard SPF conditions. Transgenic pigs (German landrace hybrid) expressing LEA29Y under control of the porcine insulin promoter were generated as described^9^. Donor piglets for transplantation experiments were produced by breeding.

### Isolation of neonatal porcine ICCs

NPICCs were isolated from 2–5-day-old piglets using a protocol described by the group of Korbutt *et al*. with some modifications^10, 11^. Briefly, pancreas was removed and placed in Hank’s Balanced Salt Solution (HBSS), cut into small pieces and digested with 1.4 mg/ml liberase (Roche, Germany) for 7–10 min at 37 °C. After filtration through a steel screen (500 μm), the tissue was washed five times with HBSS (Gibco, Germany). Then, the cell suspension was cultured in 150 mm diameter petri dishes for 5–6 days at 37 °C in RPMI 1640 (Gibco, Germany) supplemented with 2% human serum albumin (Octapharm, Germany), 10 mmol/l nicotinamide, 20 nmol/l exenatide-4 (Sigma-Aldrich, Germany) and 1% antibiotic-antimycotic (Gibco, Germany). After 5–6 days, islet clusters were harvested, washed and counted under a stereo microscope.

### Transfer of human immune cells into NSG mice

Transfer of a human immune system into NSG mice was carried out as previously described^20, 21^. NSG mice (age 6–8 weeks) were treated with busulfan (i.p. injection of 30 mg Busulfex®/kg body weight, Otsuka Canada Pharmaceuticals, Canada) to precondition the bone marrow. After one day, human CD34^+^ hematopoietic stem cells (HSC) were isolated from leukapheresis blood by ficoll density gradient centrifugation and positive selection using MACS hCD34 microbead Midi-Kit (Miltenyi Biotec, Germany) according to the manufacturer´s instruction. CD34^+^ cells with > 95% purity were transferred by tail vein injection (2.5 × 10^6^ hCD34^+^ cells per mouse). Engraftment of the human immune system was confirmed by FACS analysis of blood cells (hCD45, mCD45, hCD3) at weeks 16–20 after cell transfer. NSG mice with an established human immune system (hCD45^+^ cells > 50% in peripheral blood) were used for transplantation experiments.

### Transplantation of neonatal ICCs into hyperglycemic NSG mice

NSG mice exhibiting a robust engraftment of human immune cells (HU-SRC-SCID) or immunodeficient NSG mice (control mice for grafting) were rendered diabetic by intraperitoneal injection of 180 mg/kg streptozotocin (Sigma Aldrich, Germany). Diabetic animals (blood glucose levels > 350 mg/dl) were transplanted with either wt or LEA29Y-tg NPICCs (3000 IEQs/mouse) under the left kidney capsule as described previously^9, 11^. Neonatal NPICCs require a 6–12 weeks *in vivo* maturation period until physiological glucose-dependent insulin secretion has developed^9–11^. Blood glucose levels were monitored once per day (FreeStyle Lite, Abbott Diabetes Care, Germany). Severely diabetic mice received a daily subcutaneous insulin injection (0.25–1 IE glargine; Sanofi-Aventis, Germany) until blood glucose levels decreased to <300 mg/d. Normoglycemia was defined as persistent blood glucose levels <120 mg/dl. The observation period was set to a maximum of 125 days by study design (except of two mice, which were followed up for a period of 29 and 34 weeks post transplantation respectively). To exclude endogenous beta cell regeneration, the graft of three normoglycemic mice was removed at the end of the observation period by uninephrectomy and the reoccurrence of hyperglycemia was monitored. At predefined time-points (3 days and 3–4 weeks post-transplantation) one mouse in each group were sacrificed for immunohistochemical analyses. From each animal blood and specimen (spleen, bone marrow) for FACS analysis and immunohistochemistry (graft bearing kidney) were taken at the end of the study.

### Characterization of graft function

Intraperitoneal glucose tolerance testing (i.p.GTT) was performed 10–14 days after development of normoglycemia (BG < 120 mg/dl) using 2 g glucose/kg body weight. Blood samples were obtained from the tail vein at 0 and 10 minutes after glucose load. Porcine serum insulin was determined by ELISA (Mercodia, Sweden) that displayed no cross-reactivity with mouse insulin. Mouse C-peptide was measured by ELISA (ALPCO, USA) which had no cross-reactivity with porcine C-peptide (assay sensitivity 7.6 pmol/l, range 60–3,000 pmol/l). Plasma concentrations of LEA29Y were measured in randomly fed mice at the end of the study by sandwich ELISA as described previously^9^.

### Immunohistochemical analyses

The graft bearing kidney was subjected to immunohistochemistry to stain for insulin and infiltrating immune cells using specific antibodies for T cells (CD3, CD4, CD8, FoxP3), macrophages (CD68), and NK cells (CD56). Serial paraffin sections were stained with guinea pig anti-insulin (1:500), rabbit anti-human CD3 (1:50), mouse anti-human CD4 (1:20), mouse anti-human CD56 (1:50) (DAKO, Germany), rabbit anti-human CD8 (1:80; Vector Laboratories, USA), rabbit anti-human CD45 (1:100; DAKO, Germany), mouse anti-human CD68 (1:50, DAKO, Germany), or rabbit anti-human FoxP3 (1:50, Spring Bioscience, USA). As secondary antibodies HRP-conjugated anti-guinea pig IgG, anti-rabbit IgG, biotinylated anti-rabbit IgG or anti-mouse IgG (DAKO, Germany), and alkaline phosphatase-conjugated anti-guinea pig IgG (Southern Biotech, USA) were used. Fuchsin + Substrate (DAKO, Germany) or DAB (Kem-En-Tec Diagnostics, Denmark) were used as chromogens. To visualize CD45, CD3, CD4, and CD68 the M.O.M. Kit from Vector Laboratories was used, whereas the UltraVision™ Quanto Detection System HRP DAB (Thermo Fisher Scientific, Germany) was applied as for staining against CD8, the ImmPRESS™ Excel Amplified HRP Polymer Staining Kit (Vector) was used for detection of FoxP3, and CD56 staining was conducted with the Mouse on Mouse (M.O.M.™) ImmPRESS™ HRP (Peroxidase) Polymer Kit (Vector). Immune cell infiltration was scored independently by two researchers using insulin and anti-hCD45 co-stained sections as previously described^31^. *Beta cell insulin score*: 0: no insulin staining, 1: presence of scattered insulin-positive cells, 2: presence of insulin-positive cell aggregates, 3: presence of preserved insulin-positive clusters; *Graft immune cell infiltrate*: 0: no cell infiltrate, 1: presence of cell infiltrate in the periphery of the NPICC grafts, 2: mononuclear infiltration in the periphery and focal intra-islet infiltration, 3: extensive peri- and intra-islet infiltration. Photographs of co-stained sections against insulin and hCD3, hCD4, hCD8, hCD68, hFoxP3, or hCD56 were taken using a Leica DM2000 LED microscope coupled to a Leica CMC2900 camera (Leica Microsystems, Germany) (100x magnification). Quantitative analysis of the number of infiltrating immune cells per graft area was carried out with the Zen imaging software (Carl Zeiss, Germany). A total number of at least 5 power fields corresponding to a mean graft area of 1.5 ± 0.1 mm^2^ per animal were investigated. The frequencies of infiltrating T cells and macrophages are presented as number of infiltrating cells per mm^2^ graft tissue. The counts of hFoxP3^+^ regulatory T cells were normalized to CD4^+^ T cells and expressed in percent of CD4 + cells (number of FoxP3^+^ cells per mm^2^ graft tissue /number of CD4^+^ cells/mm^2^ graft tissue × 100).

### Flow cytometry

The proportion of human immune cells in the peripheral blood (weeks 16–20 after hCD34^+^ transfer and at the end of the study) was determined by FACS analysis. Heparinized blood (100 µl) was taken from the tail vein. After lysis of erythrocytes cells were washed in PBS with 10% FCS (FACS buffer), stained with fluorochrome-labeled monoclonal antibodies (mAb) to hCD45-APC, hCD3-PE, hCD4-APC (ebioscience, Germany), hCD8-PE (Exbio Praha, Czech Republic), mCD45-FITC (BioLegend, CA, USA), and analyzed by FACS analysis using an Accuri C6 flow cytometer (BD Biosciences, Germany). At the end of the study cell suspensions were prepared from the spleen and bone marrow as described previously^32, 33^. After lysis of erythrocytes 3 × 10^5^ cells from bone marrow or spleen were suspended in 100 µl FACS buffer and stained for 30 min with mAb to determine the frequency of murine (mCD45-FITC) and human (hCD45-APC) cells carrying leukocyte common antigen (expressed on all hematopoietic cells) and T cells (human CD3-PE, human CD4, human CD8). After two washing steps in FACS buffer cells were analyzed on Accuri C6. Matched isotype antibodies served as controls.

### Measurement of antibodies

Total human immunoglobulin M (IgM) and G (IgG) plasma levels were measured using ELISA kits (eBioscience, Germany). Xenoantibodies to the disaccharide galactosyl-alpha(1,3)-galactose (Galα1-3 Gal) epitope were measured in the sera of HU-SRC-SCID mice 7–10 days before transplantation and at the end of the observation period by ELISA. ELISA plates (Nunc/Thermo Fisher, Germany) were coated with 1 µg per well Galα1-3Galβ1-4GlcNac-HSA (3 atom pacer) (Dextra Laboratories, UK) overnight in 100 µl 100 mM carbonate buffer pH 9.4 at 4 °C. Then plates were blocked with phosphate buffered saline (PBS), pH 7.3 supplemented with 0.5% human albumin (HSA) and 0.02% Tween 20 for 2 h at room temperature (RT), washed two times with washing buffer (PBS + 0.1% HSA + 0.02% Tween 20) and incubated with 2-fold serial dilutions of the serum probes (humanized NSG mice: 1:25- 1:200; human sera: 1:250-1:5000). After 2 h plates were washed five times with washing buffer and incubated with goat anti-human IgG-HRP (1:6000) or goat anti-human IgM-HRP (1:2000) (DAKO, Germany) for 30 min at RT. Plates were washed five-times with washing buffer followed by addition of 100 µl TMB substrate solution (R&D Systems, MN, USA). After 10 min the reaction was stopped by 100 µl 2 N H_2_SO_4_. Absorbance was measured on an ELISA reader at 450 nm (Tecan, Switzerland). In each plate a standard curve (serum pool of human sera: 1:500-1:32000 for IgG α-gal antibodies and 1:100-1:8000 for IgM α-gal antibodies) and negative control sera (native NSG mice) were included. Values were expressed as arbitrary units (AU) calculated from the standard curve.

### Data presentation and statistical analysis

Data represent means and standard error of the mean (SEM). Statistical analyses were performed using the Student’s t-test or log-rank test (diabetes reversal). P values < 0.05 were considered significant.

## Electronic supplementary material

Supplementary information

## References

[1] Balamurugan AN (2014). Islet product characteristics and factors related to successful human islet transplantation from the Collaborative Islet Transplant Registry (CITR) 1999-2010. Am J Transplant.

[2] Cardona K (2006). Long-term survival of neonatal porcine islets in nonhuman primates by targeting costimulation pathways. Nat Med.

[3] Hering BJ (2006). Prolonged diabetes reversal after intraportal xenotransplantation of wild-type porcine islets in immunosuppressed nonhuman primates. Nat Med.

[4] Robertson RP (2010). Islet transplantation a decade later and strategies for filling a half-full glass. Diabetes.

[5] Shin JS (2015). Long-Term Control of Diabetes in Immunosuppressed Nonhuman Primates (NHP) by the Transplantation of Adult Porcine Islets. Am J Transplant.

[6] Thompson P (2012). Alternative immunomodulatory strategies for xenotransplantation: CD40/154 pathway-sparing regimens promote xenograft survival. Am J Transplant.

[7] Wojciechowski D, Vincenti F (2011). Challenges and opportunities in targeting the costimulation pathway in solid organ transplantation. Semin Immunol.

[8] Zhang T, Pierson RN, Azimzadeh AM (2015). Update on CD40 and CD154 blockade in transplant models. Immunotherapy.

[9] Klymiuk N (2012). Xenografted islet cell clusters from INSLEA29Y transgenic pigs rescue diabetes and prevent immune rejection in humanized mice. Diabetes.

[10] Korbutt GS (1996). Large scale isolation, growth, and function of porcine neonatal islet cells. J Clin Invest.

[11] Wolf-van Buerck, L. *et al*. Engraftment and reversal of diabetes after intramuscular transplantation of neonatal porcine islet-like clusters. *Xenotransplantation* (2015). 10.1111/xen.12201 26490671

[12] Reichart B (2015). Xenotransplantation of porcine islet cells as a potential option for the treatment of type 1 diabetes in the future. Horm Metab Res.

[13] Cardona K (2007). Engraftment of adult porcine islet xenografts in diabetic nonhuman primates through targeting of costimulation pathways. Am J Transplant.

[14] Thompson P (2011). CD40-specific costimulation blockade enhances neonatal porcine islet survival in nonhuman primates. Am J Transplant.

[15] Fu Y (2008). Selective rejection of porcine islet xenografts by macrophages. Xenotransplantation.

[16] Liu W, Xiao X, Demirci G, Madsen J, Li XC (2012). Innate NK cells and macrophages recognize and reject allogeneic nonself *in vivo* via different mechanisms. J Immunol.

[17] Zhai C (2011). Porcine CTLA4-Ig prolong islet xenografts in rats by downregulating the direct pathway of T-cell activation. Xenotransplantation.

[18] Emamaullee JA, Shapiro AM, Rajotte RV, Korbutt G, Elliott JF (2006). Neonatal porcine islets exhibit natural resistance to hypoxia-induced apoptosis. Transplantation.

[19] Korbutt GS, Warlock GL, Rajotte RV (1997). Islet transplantation. Adv Exp Med Biol.

[20] Choi B (2011). Human B cell development and antibody production in humanized NOD/SCID/IL-2Rgamma(null) (NSG) mice conditioned by busulfan. J Clin Immunol.

[21] Hayakawa J, Hsieh MM, Uchida N, Phang O, Tisdale JF (2009). Busulfan produces efficient human cell engraftment in NOD/LtSz-Scid IL2Rgamma(null) mice. Stem Cells.

[22] Tanaka S (2012). Development of mature and functional human myeloid subsets in hematopoietic stem cell-engrafted NOD/SCID/IL2rgammaKO mice. J Immunol.

[23] Kenney LL, Shultz LD, Greiner DL, Brehm MA (2016). Humanized Mouse Models for Transplant Immunology. Am J Transplant.

[24] Wu, J. *et al*. *In Vivo* Costimulation-blockade Induced Regulatory T Cells Demonstrate Dominant and Specific Tolerance to Porcine Islet-xenografts. *Transplantation* (2016). 10.1097/TP.0000000000001482 27653300

[25] Shen J (2014). Pharmacokinetics, pharmacodynamics, and immunogenicity of belatacept in adult kidney transplant recipients. Clin Drug Investig.

[26] Galili U (2013). Discovery of the natural anti-Gal antibody and its past and future relevance to medicine. Xenotransplantation.

[27] Gille C (2012). Monocytes derived from humanized neonatal NOD/SCID/IL2Rgamma(null) mice are phenotypically immature and exhibit functional impairments. Hum Immunol.

[28] Ippolito GC (2012). Antibody repertoires in humanized NOD-scid-IL2Rgamma(null) mice and human B cells reveals human-like diversification and tolerance checkpoints in the mouse. PLoS One.

[29] Klymiuk N, Ludwig B, Seissler J, Reichart B, Wolf E (2016). Current Concepts of Using Pigs as a Source for Beta-Cell Replacement Therapy of Type 1 Diabetes. Current Molecular Biology Reports.

[30] Rongvaux A (2014). Development and function of human innate immune cells in a humanized mouse model. Nat Biotechnol.

[31] Ben Nasr M (2015). Co-transplantation of autologous MSCs delays islet allograft rejection and generates a local immunoprivileged site. Acta Diabetol.

[32] Arndt B, Witkowski L, Ellwart J, Seissler J (2015). CD8 + CD122 + PD-1- effector cells promote the development of diabetes in NOD mice. J Leukoc Biol.

[33] Sattler C (2011). Inhibition of T-cell proliferation by murine multipotent mesenchymal stromal cells is mediated by CD39 expression and adenosine generation. Cell Transplant.

