# Supplementary material for: LEA29Y expression in transgenic neonatal porcine islet-like cluster promotes long-lasting xenograft survival in humanized mice without immunosuppressive therapy
Source: Sci Rep. 2017 Jun 15;7:3572. doi: 10.1038/s41598-017-03913-4 (PMC5472587; doi:10.1038/s41598-017-03913-4)
Supplement: Supplementary file 1 — Supplementary information [file 41598_2017_3913_MOESM1_ESM.pdf]

LEA29Y expression in transgenic neonatal porcine islet-like cluster promotes long-lasting xenograft survival in humanized mice without immunosuppressive therapy

L. Wolf-van Buerck, M. Schuster, F.S. Oduncu, A. Baehr, T. Mayr, S. Guethoff, J. Abicht, B. Reichart, N. Klymiuk, E. Wolf, J. Seissler

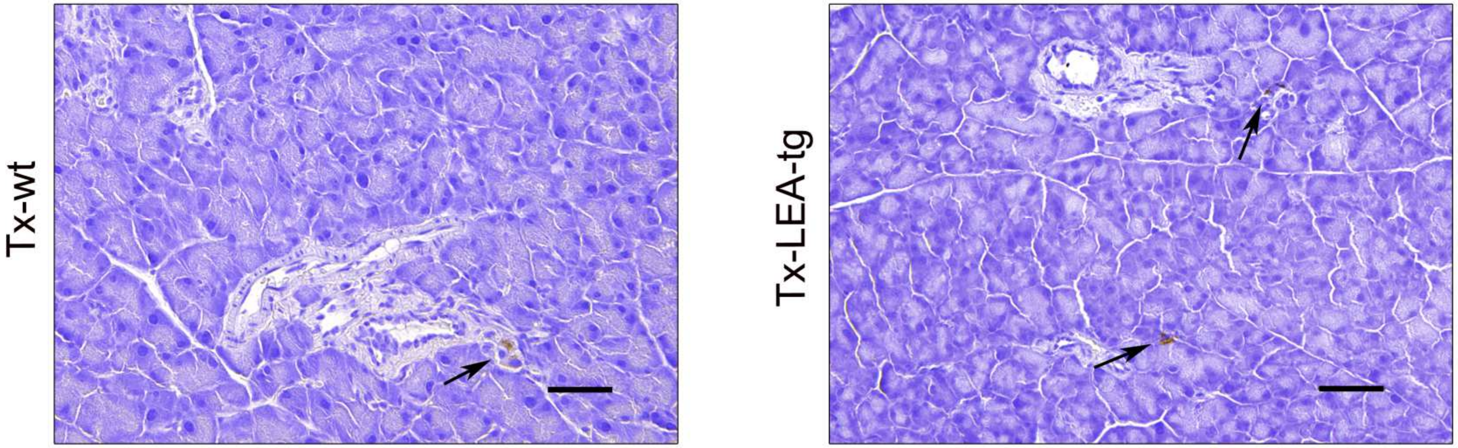

Supplemental figure 1: Immunohistochemical staining of beta cells in recipient pancreata.

Immunohistochemical analysis of pancreata from mice transplanted with wild-type (Tx-wt) or *INS*LEA29Y transgenic (Tx-LEA-tg) NPICCs at the end of the study revealed only singular if any insulin^{+} (brown) stained beta cells (marked by an arrow) excluding endogenous beta cell regeneration.

Scale bar: 100 µm.

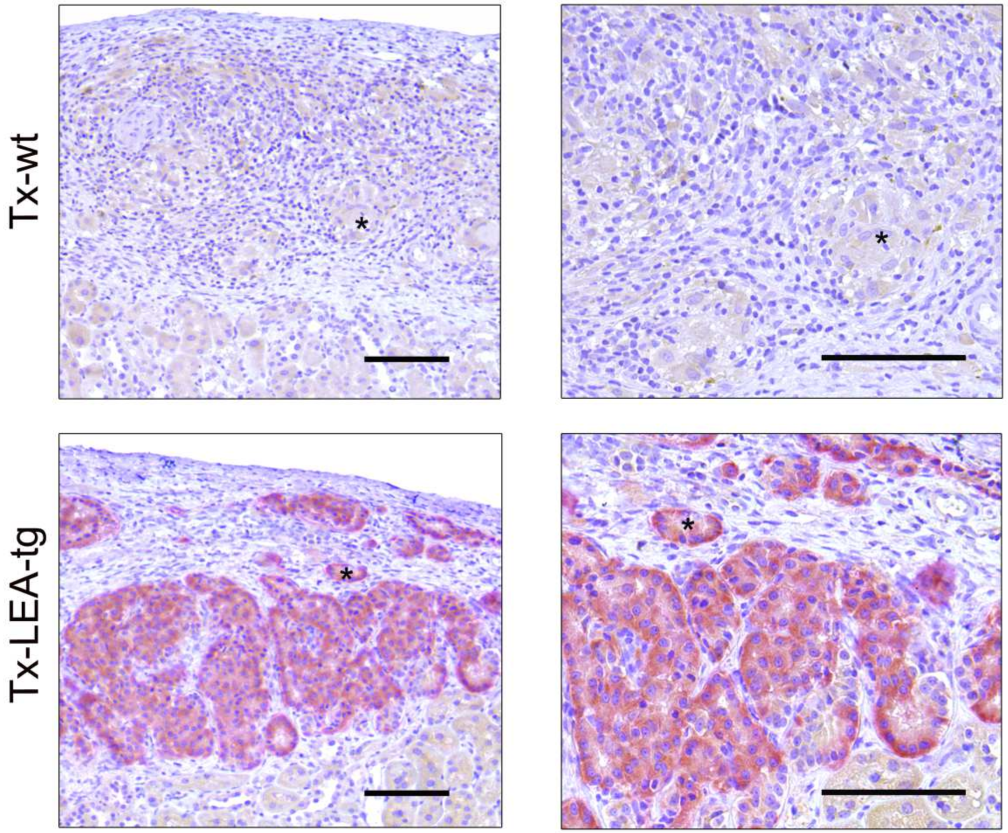

Supplemental figure 2: CD56⁺ NK cells are not detectable in NPICC grafts.

Immunohistochemical staining of NPICC grafts against the NK cell marker hCD56 (brown) and insulin (red) (left: 100x magnification, right: 200x magnification – identical positions are marked by an asterisk) did not reveal a specific signal for CD56 in grafts of both, mice transplanted with wild-type (Tx-wt) or *INS*LEA29Y transgenic (Tx-LEA-tg) NPICCs.

Scale bar: 100 µm.

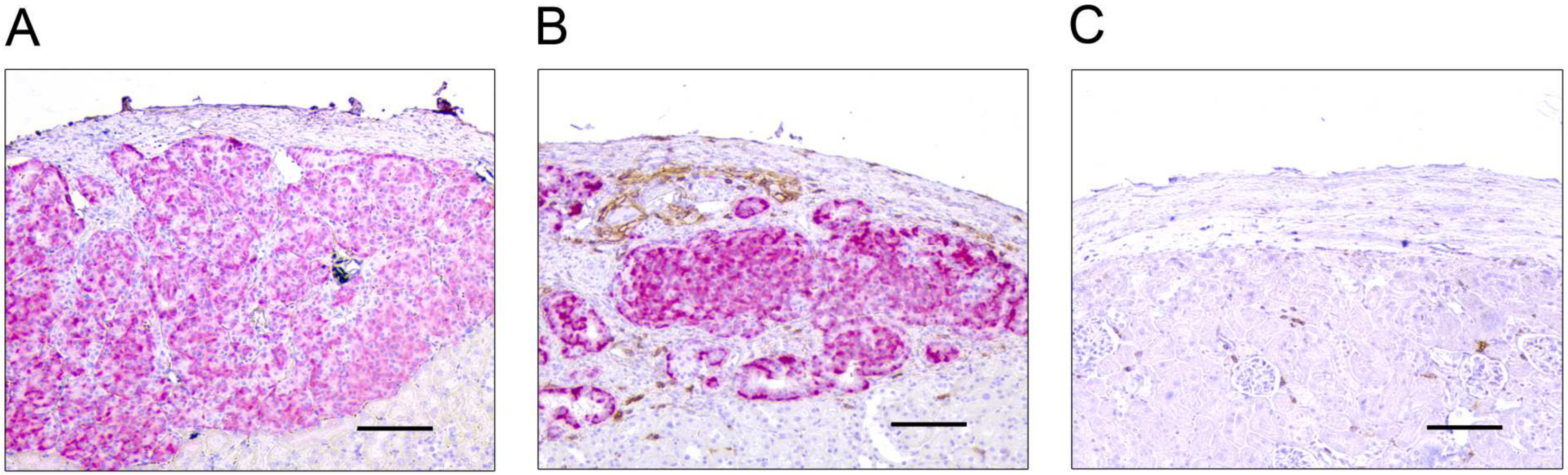

Supplemental figure 3: Graft morphology in non-normoglycemic mice transplanted with LEA29Y transgenic NPICCs.

In two mice with LEA29Y transgenic NPICCs that developed almost normal blood glucose levels (A, B), the subcapsular grafts exhibits an intact morphology with widely insulin^{+} (red) stained endocrine tissue and only mild infiltration with hCD45^{+} lymphocytes (brown). The graft of the single mouse that failed to improve hyperglycemia exhibited few CD45^{+} stained cells but no detectable beta cells (C).

Scale bar: 100 µm.
